# New Evidence for *Artemisia absinthium* L. Application in Gastrointestinal Ailments: Ethnopharmacology, Antimicrobial Capacity, Cytotoxicity, and Phenolic Profile

**DOI:** 10.1155/2021/9961089

**Published:** 2021-07-22

**Authors:** Marija Ivanov, Uroš Gašić, Dejan Stojković, Marina Kostić, Danijela Mišić, Marina Soković

**Affiliations:** Department of Plant Physiology, Institute for Biological Research “Siniša Stanković”—National Institute of Republic of Serbia, University of Belgrade, Bulevar Despota Stefana 142, Belgrade 11000, Serbia

## Abstract

*Artemisia absinthium* L. (Asteraceae) is traditionally used for gastrointestinal ailments and disorders linked to numerous risk factors including microbial infections. We aimed to provide contemporary evidence for its ethnopharmacological use and determine its antimicrobial capacity and mode of action, cytotoxicity, and phenolic constituents. Ethnopharmacological survey was conducted using semistructured interviews. Antimicrobial and antibiofilm capacities were determined by microdilution/crystal violet assay, respectively. Modes of action tested include estimation of exopolysaccharide production (congo red binding assay) and interference with membrane integrity (crystal violet uptake and nucleotide leakage assay). Cytotoxicity was determined using crystal violet assay. Polyphenolic profiling was done by advanced liquid chromatography/mass spectrometry (UHPLC-LTQ OrbiTrap MS). *Artemisia absinthium* in Serbia is traditionally used for gastrointestinal disorders, among others. Further study revealed high antifungal capacity of herb ethanolic extract towards range of *Candida* species (MIC 0.5–1 mg/mL) along with promising antibacterial activities (MIC 0.25–4 mg/mL). Interference with membrane integrity could be observed as a possible antimicrobial mechanism. Antibiofilm potential can be considered as high (towards *C. krusei*) to limited (towards *P. aeruginosa*) and moderate based on reduction in exopolysaccharide content. In concentrations up to 400 *µ*g/mL, no cytotoxicity was observed towards HaCaT and HGF-1 cell lines. Polyphenolic analysis revealed twenty-one different constituents. *A. absinthium* usage as a gastrointestinal ailment remedy has been confirmed *in vitro* by its antimicrobial capacity towards microorganisms whose presence is linked to the diseases and associated complications and noncytotoxic nature of the natural product. The observed activities could be attributed to the present phenolic compounds.

## 1. Introduction

*Artemisia absinthium* L. (Asteraceae), wormwood, is perennial shrubby medicinal plant. This plant is used in shampoos, face serums, masks, essences, and other cosmetology products along with abundant utilization in food industry as the main flavoring ingredient in alcoholic beverage absinth [1]. The essential oil obtained from this species is rich in bioactive chemical constituents such as *cis*-epoxy-*o*-cimene, chrysanthenol, and chrysanthenyl acetate [2] while herb extracts are rich sources of different biomolecule classes such as flavonoids, coumarins, and fatty acids [1].

Its aerial parts are traditionally used worldwide for digestive discomforts and gastrointestinal (GI) ailments, and due to antimicrobial and diuretic properties [3] with randomized controlled trials confirming its bioactivity in Crohn's disease [4]. Ranges of microorganisms are found to have a role in the Crohn's disease etiology including Streptococci, *Pseudomonas aeruginosa*, and *Listeria monocytogenes* [5]. Patients suffering from Crohn's disease and other GI ailments are also more frequently colonized by *Candida* spp. compared to healthy individuals [6].

The GI disease presence is being linked to a range of factors including chronic sinonasal diseases [7] with tonsillectomies seen as a risk factor for inflammatory bowel disease [8]. The link between oral disease and GI ailments is the ability of bacteria to translocate from oral cavity into the GI tract by hematogenous and enteral routes giving the opportunity for oral microbes to cause a range of GI diseases [9]. Even pathogens, such as *Klebsiella pneumoniae,* which were until recently referred to mainly as upper respiratory tract colonizers, have now emerged as notable GI tract-associated microorganisms [10]. Biofilm forming abilities of pathogenic microorganisms have been linked to the onset of GI diseases such as colorectal cancer [11] and inflammatory bowel disease [12].

Our study aimed to investigate the ethnopharmacological use of wormwood among Serbian population and to provide modern evidence for the uses currently practiced in Serbia. Therefore, this study aimed to examine the effects of *A. absinthium* ethanol extract on a range of pathogens associated with GI disorders as well as their impact on biofilm forming abilities of microbial species. Chemical analysis was performed in order to reveal bioactive constituents of the medicinal herb.

## 2. Materials and Methods

### 2.1. Ethnopharmacological Survey on the Use of *A. absinthium*—Current Update

The survey was performed using semistructured questionnaires via a face-to-face interview and circulating these questionnaires among cross section of people above 20 years of age; 117 filled-up reports were collected throughout Serbia. A questionnaire in Serbian language was prepared about the use of *Artemisia absinthium* L. (Asteraceae) by the local people from different parts of Serbia, as described previously for the plant species [13]. All the respondents were aware of the present investigation and have signed the informed consent. The survey was conducted during three months. The survey covered different age groups of both sexes, whose gender, age, educational background, professional status, and knowledge on the use of *A. absinthium* were also documented. Each participant was interviewed separately to generate data on diseases, regarding the treatment through medicinal plant *A. absinthium*. The record of questionnaires used included the following information: (a) the local name, (b) part of the plant used, (c) method of preparation, (d) mode of application, and (e) ethnomedicinal uses. Fidelity level (FL) was applied for diseases or ailments that were reported [13]. It is a ratio of informants claiming the use of a plant species for a particular purpose (Np) and number of informants using the plant to treat any disease (*N*). It was calculated by the formula

(1) $$\begin{array}{c} \text{FL}\%=\text{Np}/N\times 100. \end{array}$$

### 2.2. Chemicals and Materials

Acetonitrile and formic acid (both of analytical grade) were purchased from Merck (Darmstadt, Germany). Analytical standards of phenolic compounds (chlorogenic acid, esculin, rutin, narcissin, isorhamnetin-3-O-glucoside, rosmarinic acid, apigenin, chrysoeriol, and kaempferide) were supplied by Sigma-Aldrich (Steinheim, Germany).

### 2.3. Preparation of the Extract

Plant *Artemisia absinthium* was collected on Ramski pesak, Serbia, during 2017. Samples of the collected plant material were identified based on the scientific botanical literature and its morphological features by one of the authors (D.S.). The plant species was deposited in our local institutional herbarium under number MI2017/Aa. A plant sample was air-dried and cut into small pieces; 10 g of the plant herb was extracted using 300 mL of ethanol (absolute, for analysis, Merck, Germany) at −20°C overnight. The extract was sonicated for 15 min and filtered through the Whatman No. 4 paper. The plant residue was reextracted using another 300 mL of ethanol, and the procedure was repeated. The obtained ethanol extract was evaporated at 40°C on a rotary evaporator (Büchi R-210) to dryness, and the dried extract was dissolved in 30% ethanol (Merck, Germany).

### 2.4. Antimicrobial Activity

#### 2.4.1. Microorganisms and Culture Conditions

*Candida* species used were clinical isolates *C. albicans* 475/15, *C. albicans* 13/15*, C. albicans* 17/15*, C. krusei* H1/16, and *C. glabrata* 4/6/15 that were obtained and maintained as described in [14]. Reference yeast strains used were *C. albicans* ATCC 10231, *C. tropicalis* ATCC 750, and *C. parapsilosis* ATCC 22019.

The following Gram-positive and Gram-negative clinical bacteria were used: *Micrococcus luteus* (dT_9/2), *Rothia mucilaginosa* (oT_22/2), *Streptococcus agalactiae* (oT_20/1), *Streptococcus anginosus* (oT_26), *Streptococcus dysgalactiae* (oT_21/2), *Streptococcus oralis* (oT_5), *Streptococcus parasanguinis* (oT_3), *Streptococcus pyogenes* (dT_14), *Streptococcus pyogenes* (IBR S004), *Streptococcus salivarius* (dT_12), *Streptococcus salivarius* (IBR S006), *Staphylococcus hominis* (oT_14/2), *Enterococcus faecalis* IBRE001, *Enterobacter cloacae* (oT_18), and *Stenotrophomonas maltophilia* (A_12) obtained and maintained as described previously [15, 16].

Resistant strains used were *Pseudomonas aeruginosa* (IBRS P001), methicillin-resistant *Staphylococcus aureus* (IBRS MRSA 011), and *Escherichia coli* (IBRS E003) previously described by Victor et al. [17].

Reference bacterial American Type Culture Collection strains used were *Listeria monocytogenes* (NCTC 7973), *Yersinia enterocolitica* (ATCC 23715), *Klebsiella pneumoniae* (ATCC 13883), *Escherichia coli* (ATCC 35210), *Salmonella enterica* (ATCC 13311), and *Enterobacter cloacae* (ATCC 35030).

Tested microorganisms are deposited at the Mycological Laboratory, Department of Plant Physiology, Institute for Biological Research “Siniša Stanković”—National Institute of Republic of Serbia, University of Belgrade.

#### 2.4.2. Anticandidal Activity

Minimal inhibitory and minimal fungicidal concentration (MIC/MFC) were determined [18]. Briefly, fresh overnight yeast cultures were adjusted to a concentration 1.0 × 10^5^ CFU/well with the use of sterile saline. The microplates were incubated at 37°C for 24 h, after which the MIC and MFC were determined. The MIC values were considered as the lowest concentrations without microscopically observed growth. Following the serial subcultivations of 10 *μ*L into microtiter plates containing 100 *μ*L of broth per well, as well as subsequent incubation at 37°C for 24 h, the lowest concentrations with no visible growth were defined as the MFC values, indicating 99.5% killing of the original inoculum. Ketoconazole was used as a positive control (Sigma-Aldrich, Germany).

#### 2.4.3. Antibacterial Activity

Minimum inhibitory concentrations (MIC) and minimum bactericidal concentrations (MBC) were determined by a serial microdilution of *A. absinthium* extract in 96-well microtiter plates following the protocol described by Kostić et al. [19]. Streptomycin was used as positive control.

#### 2.4.4. Inhibition of Biofilm Formation

Strains used for inhibition of biofilm formation assay were *C. albicans* 475/15, *C. krusei* H1/16, *Klebsiella pneumoniae* (ATCC 13883), *Streptococcus pyogenes* (IBR S004), and *Pseudomonas aeruginosa* (IBRS P001). Impact of plant extract on microbial attachment as the first stage of biofilm formation was determined as described by Smiljković et al. [20]. Microorganisms were incubated for 24 h in 96-well microtiter plates with an adhesive bottom (Sarstedt, Germany) at 37°C with MIC and sub-MIC concentrations of the compound. After 24 h, each well was washed twice with sterile PBS (phosphate-buffered saline, pH 7.4). Fixation of adhered cells was done with methanol, after which the plate was air-dried and stained with 0.1% crystal violet (BioMérieux, France) for 30 min. Wells were washed with water, air-dried, and then 100 *μ*L of 96% ethanol (Zorka, Serbia) was added into wells to suspend all the bound stain. Absorbance was read at 595 nm with a Multiskan FC Microplate Photometer (Thermo Scientific). Percentage of inhibition of biofilm formation was calculated by using the following equation:

(2) $$\begin{array}{c} \left[\frac{\left(\text{A}595\text{ control}-\text{A}595\text{ sample}\right)}{\text{A}595\text{ control}}\right]\times 100. \end{array}$$

#### 2.4.5. Congo Red Binding Assay

The impact of tested compound on exopolysaccharide (EPS) production by *C. albicans* 475/15 biofilm was estimated with some modifications according to the method previously published [21]. Preformed 24 h biofilms in microtiter plates were treated with *A. absinthium* extract at its MIC, 0.5 MIC, and 0.25 MIC concentrations for 24 h at 37°C. Planktonic cells were then discarded and the adhered cells were washed with PBS. Congo red (1%, w/v) was added to wells and was kept in dark for 30 min. Excess dye was removed and the bound congo red was solubilized with 200 *μ*L DMSO. The absorbance was measured at 490 nm in a microtiter plate reader. Percentage of EPS inhibition was calculated according to the following equation:

(3) $$\begin{array}{c} \%\text{inhibition}=\frac{\left({\text{OD}}_{490}\left(\text{control}\right)-{\text{OD}}_{490}\left(\text{sample}\right)\right)}{{\text{OD}}_{490}\left(\text{control}\right)}*100. \end{array}$$

#### 2.4.6. Crystal Violet Uptake Assay

Alteration of membrane permeability was detected by modified crystal violet uptake assay [22]. *C. albicans* 475/15 cells were harvested by centrifugation 10,000 rpm for 5 min, washed twice, and suspended in 50 mM PBS (pH 7.4). Plant extract in its previously determined minimal inhibitory concentration was added to the cell suspension, and samples were incubated at 37°C for 30 min. Control samples were untreated *C. albicans* cells. The cells were harvested at 10,000 rpm for 5 min and suspended in PBS containing 10 *μ*g/mL of crystal violet. The cell suspension was incubated for 10 min at 37°C and centrifuged at 10,000 rpm for 5 min, after which the OD_595_ of the supernatant was measured using a Multiskan FC Microplate Photometer (Thermo Scientific). The optical density (OD) value of crystal violet solution, which was originally used in the assay, was considered as 100% excluded. The percentage of crystal violet uptake was calculated following the formula

(4) $$\begin{array}{c} \%\text{dye uptake}=100-\left[\left(\frac{\text{OD of the sample}}{\text{OD value of crystal violet solution}}\right)\times 100\right]. \end{array}$$

#### 2.4.7. Nucleotide Leakage Assay

The impact of *A. absinthium* extract on fungal membrane permeability (nucleotide leakage) was determined according to previously published protocol [23, 24] with some modifications and compared to untreated yeast cells. The culture of *C. albicans* 475/15 incubated overnight at 37°C was washed twice and resuspended in 10 mM PBS (pH 7.4), reaching the final density of 10^8^ CFU/mL. Strain was incubated with the extract at the MIC for 30 min; *C. albicans* incubated with 10 mM PBS (pH 7.4) was used as control. After incubation, cell suspensions were centrifuged at 10,000 g for 10 min and supernatant absorbance measured at 260 nm and 280 nm with Agilent/HP 8453 UV-Visible Spectrophotometer (Agilent Technologies, USA) at room temperature (25°C).

### 2.5. Cytotoxicity

Cytotoxic effect of *A. absinthium* alcoholic extract was determined on human gingival fibroblasts cells (HGF-1) and spontaneously immortalized keratinocyte cell line (HaCaT) using crystal violet assay as described previously [25]. The extract of *A. absinthium* was dissolved in PBS to a final concentration of 8 mg/mL. HGF-1 cells were grown in Fibroblast Basal Medium (ATCC^®^ PCS-201-030™), while HaCaT cells were grown in high-glucose Dulbecco's Modified Eagle Medium (DMEM) supplemented with 10% fetal bovine serum (FBS), 2 mM L-glutamine, and 1% penicillin and streptomycin (Invitrogen), at 37°C in a 5% CO_2_ incubator. Forty-eight hours before treatment, cells were seeded in a 96-well microtiter adhesive plate at a seeding density of 4 × 10^3^ cells per well. After 48 h, the medium was removed and the cells were treated for next 24 h with various concentration of the extract in triplicate wells. Subsequently, the medium was removed; the cells were washed twice with PBS and stained with 0.4% crystal violet staining solution for 20 min at room temperature. Afterwards, crystal violet staining solution was removed; the cells were washed in a stream of tap water and left to air dry at room temperature. The absorbance of dye dissolved in methanol was measured in a plate reader at 570 nm (OD_570_). The results were expressed as relative growth inhibition (GI_50_) rate (%).

### 2.6. UHPLC-LTQ OrbiTrap MS Analysis of Polyphenolic Compounds

Separation and identification of polyphenols in tested extract were done by using UHPLC system (Accela 600) coupled to LTQ OrbiTrap MS (Thermo Fisher Scientific, Bremen, Germany). All the method details about chromatographic separation and setting of mass detector were given in previous work [26].

Some compounds were confirmed by comparison with the appropriate standard, while other compounds were tentatively identified by high resolution mass spectrometry (HRMS) and MS^n^ fragmentation using appropriate literature about analysis of various *Artemisia* species [27–34].

### 2.7. Statistical Analysis

All the experiments were performed in three repeats. All the data were calculated as a mean ± standard error and statistically analyzed using GraphPad PRISM 6 software.

## 3. Results and Discussion

### 3.1. Ethnopharmacological Investigation on *A. absinthium* Use among People in Serbia

The results of ethnopharmacological survey are presented in Table 1. A total of 117 informants were interviewed. Among them, 30 were residing in northern Serbia, 25 in east Serbia, 24 in central Serbia, 21 in west Serbia, and 17 in southern Serbia. Majority of informants (67.52%) were females, while the most represented age group was between 40 and 60 years (57.26%), with secondary educational background (47.86%). Among 117 informants, 29 reported no knowledge of *A. absinthium* neither in ethnobiology as a food or drink nor in medicinal purposes. On the other hand, 88 informants reported 138 medicinal uses of *A. absinthium*. The most prevalent use of *A. absinthium* was as aperitif or appetizer in alcoholic preparations. With a fidelity level of 39.77%, GI disorders were highly ranked reported use among the local people (mostly frequently alcoholic preparations and rarely tea), followed by the use of *A. absinthium* in wound-healing (laying the plant directly on wounds or preparations based on mixture of powdered plant material and animal fat). Other uses were less frequently reported (Table 1). Our ethnopharmacological survey conducted among the people living in Serbia is in accordance with previous literature data published on the use of *A. absinthium* [3, 35]. Since one of the most frequent uses for wormwood in Serbia was the application to improve the state of GI ailments, we strived to enlighten the activity of alcoholic extract against bacteria and fungi linked to GI disorders.

### 3.2. Antimicrobial Capacity of *A. absinthium* L. and Insights into the Modes of Action

The examined extract has shown promising antifungal properties with MIC in the range 0.5–1 mg/mL. Moderately higher resistance could be observed for non-albicans *Candida* strains *C. krusei* and *C. glabrata* with MIC 1 mg/mL (Table 2).

A previous study of *A. absinthium* essential oil [36] has indicated GIC_50_ (growth inhibitory concentration for 50% of microorganisms) 0.1 mg/mL, indicating better activity than the one recorded for the extract in our study. Aqueous plant extract tested at maximal 0.5 mg/mL concentration was ineffective against *Candida parapsilosis* ATCC 22019 and *Candida albicans* ATCC 90028 [37]. The study by Valdes et al. [38] found IC_50_ of ethanol extract towards *C. albicans* to be higher than 64 *µ*g/mL. Study of apigenin [23], a compound present in the *A. absinthium* extract (Table 3), determined MIC towards different *Candida* species in the range 0.1–0.15 mg/mL, while another constituent, rutin, exhibited MIC 0.0375 mg/mL [39], indicating it as a possible carrier of antifungal activity.

The antifungal potential of *A. absinthium* ethanolic extract determined in our study is a solid basis for further antifungal development and provides a scientific evidence for the traditional usage of *A. absinthium* for GI disorders linked to fungal overgrowth. Species belonging to the genus *Candida* are residing on the mucosal surfaces of different parts of gastrointestinal tract including oral mucosa and gut [40]. However, this colonization is not always harmless, and these fungal species can cause severe infections. Besides oral candidiasis, these yeasts are also the most common cause of the infectious esophagitis [41]. *C. glabrata* had also been linked with Crohn's disease, where it possibly induces gut inflammation [42], while the presence of *C. krusei* in the stomach has been associated with gastritis and ulcers [43].

Ethanolic extract of *A. absinthium* herb has shown excellent antibacterial potential with MIC 0.25–4 mg/mL (Table 4). The most susceptible to the extract was *Streptococcus salivarius* (dT_12) with MIC 0.25 mg/mL, while the most resistant were *L. monocytogenes* (NCTC 7973) and resistant strains of *E. coli* (IBRS E003) and *S. aureus* (IBRS MRSA 011) with MIC 4 mg/mL. Resistant strain of *P. aeruginosa* (IBRS P001) was susceptible to the treatment with 1 mg/mL of herb extract.

A recent study [44] of *A. absinthium* methanol extract has indicated MIC 2.5–1.255 mg/mL towards *Escherichia coli* ATCC 10536, while in our study it exhibited MIC 4 mg/mL towards *E. coli* (IBRS E003) resistant strain. Unlike high susceptibility of resistant *P. aeruginosa* strain recorded in our study, in the study by Boudjelal et al. [44], it did not show activity (MIC > 2.5 mg/mL) as well as the one by Khan et al. [45]. Methanolic extract studied previously by Hasannezhad et al. [46] has shown MIC 41.7 mg/mL towards *L. monocytogenes* PTCC 1298, unlike 4 mg/mL determined in our study. Methanol extract concentration 8 mg/mL reduced *Escherichia coli* ATCC 25922 growth for 51.5% [47].

The ethanol extract from *A. absinthium* growing wild in Serbia has shown promising antibacterial potential towards a range of bacterial strains with eight of them having MIC ≤ 0.5 mg/mL. Range of bacterial species is nowadays associated with gastrointestinal ailments. Likewise, antibiotic-resistant strains of *P. aeruginosa* are frequent colonizers of intensive care units patients' GI tract [48], while colons of Crohn's disease patients have higher frequency of *E. coli* [49]. On the other hand, increased levels of *Streptococcus* species have been associated with upper gastrointestinal symptoms of functional dyspepsia [50]. Recently, *K. pneumoniae* has been highlighted as the frequent GI disease-associated pathogenic bacterium being linked to the diseases such as Crohn's disease, ulcerative colitis, and colorectal cancers [10].

**Figure 1:**
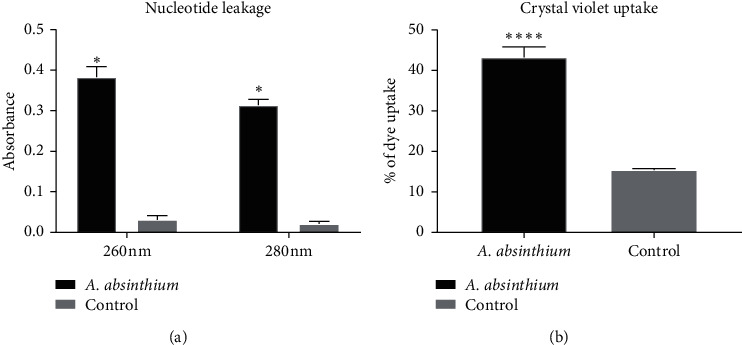
Destabilization of *C. albicans* 475/15 membrane after treatment with *A. absinthium* (MIC, 0.5 mg/mL) for 30 min detected by (a) crystal violet uptake (%) and (b) leakage of nucleic acids (260 nm) and proteins (280 nm). The error bars indicate standard deviations. The asterisks represent statistical significance (^*∗*^*p* < 0.05; ^*∗∗∗∗*^*p* < 0.0001).

Strain *C. albicans* 475/15 was selected as the reference strain for the investigations of antimicrobial mode of action. It can be observed that application of *A. absinthium* significantly affected membrane integrity (Figure 1) as observed by dramatic increase in the uptake of crystal violet (Figure 1(a)) and further confirmed by an increase in nucleotide and protein leakage (Figure 1(b)).

Previous studies conducted with apigenin, the phenolic compound present in *A. absinthium* (Table 3), proved the membrane antagonistic activity for single extract constituent [23], while the study of *A. asiatica* essential oil proved its potential to induce leakage of cell constituents [51].

Ability of the microorganisms to group into biofilms has been associated with numerous chronic diseases including those of GI tract [52]. Likewise, *E. coli* ability to establish biofilms has been linked to the presence of ulcerous colitis [53]. It has been shown for *C. albicans* that genes associated with its adhesion ability, which is the first stage of biofilm formation, have higher expression levels during the colonization of the cecum and invasion of host tissue [54].

**Figure 2:**
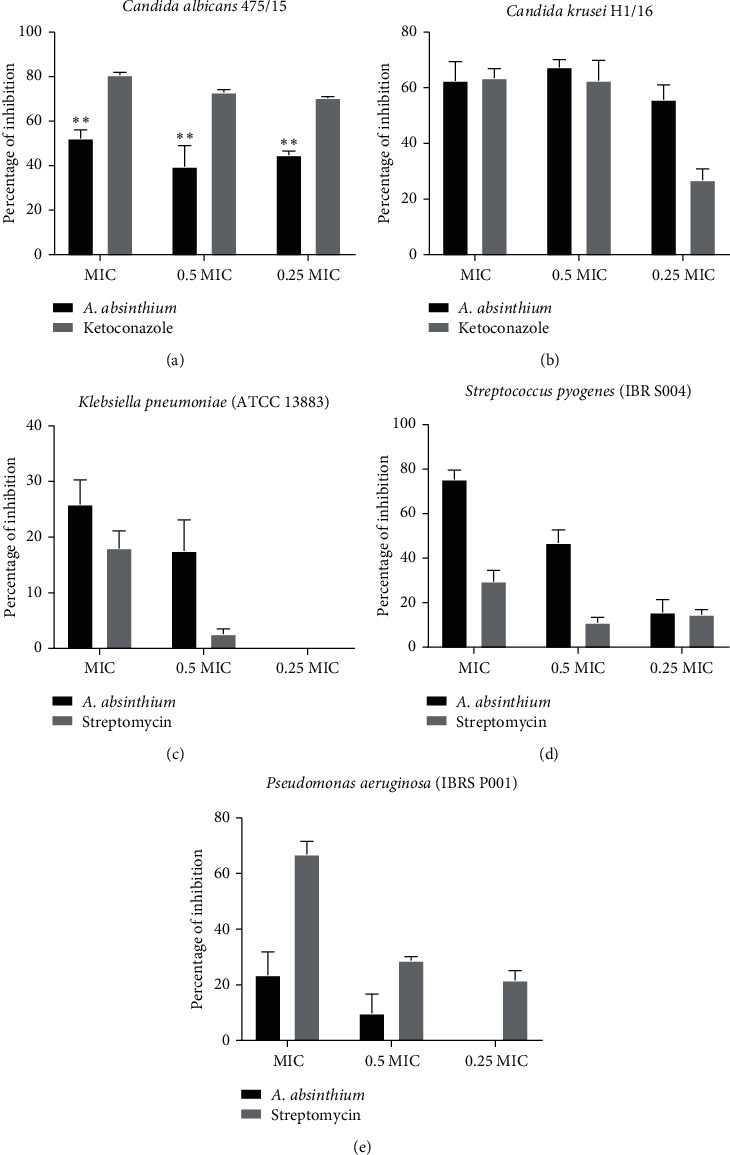
Percentage of inhibition of fungal and bacterial biofilm formation after treatment with *A. absinthium* in range of concentration 0.125 mg/mL–1 mg/mL. The error bars indicate standard deviations. The asterisks represent statistical significance (^*∗∗*^*p* < 0.05).

Extract of *A. absinthium* has shown a promising potential in the means of reduction of microbial ability to establish biofilms (Figure 2). Application of extract at 0.5 MIC concentration has reduced *C. krusei* biofilm formation ability for more than 50%, with the significant reduction (>50%) observed also for MIC of extract towards *C. albicans* 475/15 and *S. pyogenes* IBR S004. The least promising was its ability to interfere with *P. aeruginosa* (IBRS P001) and *K. pneumoniae* ATCC 13883 biofilms since it caused less than 30% reduction with the highest applied concentration (MIC).

Plant extracts tested by Khan et al. [45] were not able to interfere significantly with *S. aureus* biofilm, while the investigation of caffeoylquinic acids from *A. absinthium* proved their antibiofilm effect towards *S. aureus* and *E. faecalis* [55].

**Figure 3:**
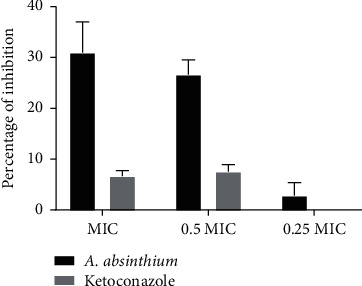
Estimated inhibition (%) of exopolysaccharide biofilm matrix content by congo red binding assay determined using *C. albicans* 475/15 biofilm after treatment with *A. absinthium* compared to commercial antifungal drug ketoconazole. The error bars indicate standard deviations. Statistical difference was not significant.

The mode of antibiofilm mechanism was investigated on the strain *C. albicans* 475/15 in order to establish whether reduction in exopolysaccharide production by the fungal cells is the reason for observed reduced biofilm capacity in previous experiment (Figure 2). Treatment with MIC of *A. absinthium* extract has reduced the exopolysaccharide bound congo red for more than 25% indicating that it might be moderately involved as the plant antibiofilm mechanism (Figure 3).

### 3.3. Cytotoxicity

**Figure 4:**
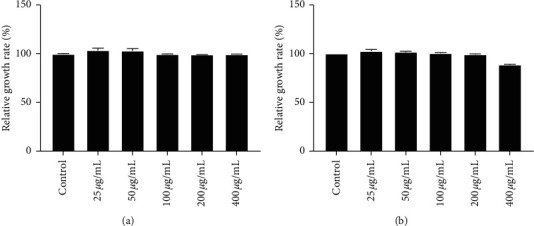
Cytotoxicity of *A. absinthium* alcoholic extract towards (a) HGF-1 cells and (b) HaCaT cell line, represented as relative growth rates (%) at different concentrations of the extract. The error bars indicate standard deviations among three independent replicates.

Cytotoxicity of *A. absinthium* extracts is demonstrated in Figure 4. It could be observed that growth rate of both cell lines was not significantly affected by *A. absinthium* up to a concentration 400 *µ*g/mL. The concentration 400 *µ*g/mL had only effect on HaCaT cell line where growth rate was 85.47% when compared to the control. The previous studies point that concentration that caused decrease in growth rate for 50% and higher than 401 *µ*g/mL should be considered as nontoxic [25].

Previous study of the leaves and stem ethanolic extracts [56] showed reduction in HaCaT cell viability (71.6% viability with 1000 *µ*g/mL of extract) while range of concentrations as the one tested in our study (25–400 *µ*g/mL) did not cause significant reduction in cell viability. Although a different methodology was used (alamarBlue assay by Moacă et al. [56] and crystal violet assay in our study), results prove noncytotoxic nature of the tested plant extract. Previous study of ethanolic extract of *Artemisia apiacea* proved its noncytotoxicity towards HaCaT in a concentration up to 200 *µ*g/mL [57].

### 3.4. Chemical Profiling

This study describes the UHPLC-LTQ OrbiTrap MS polyphenolic profile of the *Artemisia absinthium* extract. A total of 21 compounds (Table 3) were identified. Nine compounds were identified using analytical standards, while the other twelve were identified by the search for their [M–H]^−^ deprotonated molecules combined with its MS^4^ fragmentations.

Examination of mass spectra revealed seven phenolic acid derivatives. The most represented compounds from this group were quinic acid derivatives: chlorogenic acid (**3**), feruloylquinic acid (**5**), two derivatives of dicaffeoylquinic acid (**11** and **13**), and rosmarinic acid (**16**).

**Figure 5:**
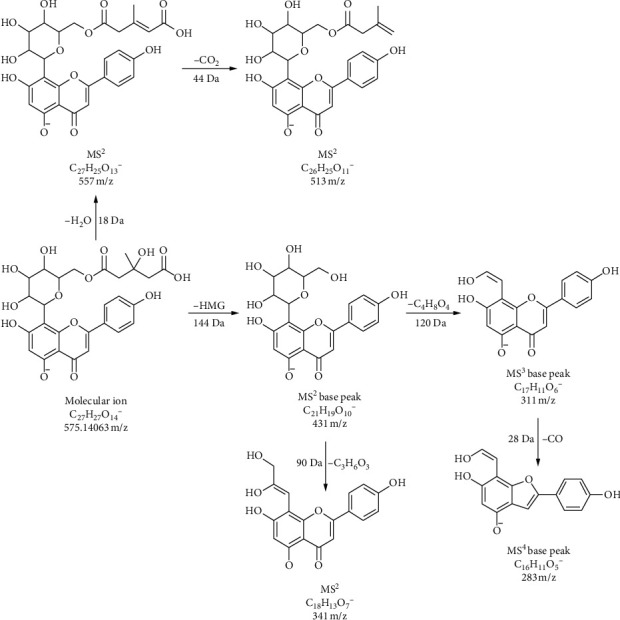
Fragmentation pathway of compound 9 (apigenin 8-C-[6“-(3-hydroxy-3-methylglutaryl)]hexoside).

Eight compounds from the group of flavonoid glycosides were identified. Four of them (**6**, **7**, **8**, and **10**) were marked as rutinosides, because as MS^2^ base peak they give a fragment ion formed by the loss of 308 Da (rhamnosyl-hexoside). Fragmentation pathway of kaempferol 7-O-(6″-rhamnosyl) hexoside (compound **7**) was confirmed in literature [58]. It should be noted here that kaempferol 3-O-(6″-rhamnosyl)hexoside has been previously identified in some *Artemisia* species [27], but in our case the fragmentation corresponds to a 7-O derivative [58]. Namely, the position of sugar binding to flavonoids can be sensed by the abundance of the molecular ion of aglycone and their radical ion [59]. Of course, for accurate confirmation of this claim, it is necessary to isolate this compound and record its NMR spectra. Compounds **12**, **14**, and **15** were identified as hexosides of isorhamnetin, spinacetin, and chrysoeriol, respectively. The MS^2^ base in the case of these three compounds is formed by a neutral loss of 162 Da. Apigenin 8-*C*-[6″-(3-hydroxy-3-methylglutaryl)]hexoside (**9**) is a compound that, in our opinion, has not yet been identified in *Artemisia* species. In the first fragmentation step, this compound loses the hexosyl group (162 Da) and thus the MS^2^ base peak is formed. The MS^3^ base peak is generated by homolytic sugar cleavage (120 Da) and further neutral loss of 28 Da (CO) gives MS^4^ base peak. The detailed fragmentation pathway of this compound is depicted in Figure 5. Confirmation that aglycone part of this compound was vitexin (apigenin 8-*C*-glucoside) and not isovitexin (apigenin 6-*C*-glucoside) has been found in the literature [60].

Considering the five identified flavonoid aglycones, three were confirmed by comparison with standards (**17**, **18**, and **21**) and two (**19** and **20**) were tentatively identified by examination of its MS spectra. Compound **19** was previously detected in aerial parts of *Artemisia incisa* Pamp [61], while compound **20** was identified in the crude extracts and some fractions of *Folium Artemisia* Argyi, a traditional Chinese herb medicine and food supplement [33].

## 4. Conclusions

*Artemisia absinthium* L. wide usage as a traditional remedy for GI diseases might be based on the ability of the herb to reduce the growth of microorganisms whose presence is linked to GI discomfort. This antimicrobial potential could be attributed to the broad spectrum of bioactive phenolic compounds present in the herb ethanol extract since for some of them identical mechanism of antimicrobial activity and wide antimicrobial spectrum has been determined previously.

## Figures and Tables

**Table 1:** Ethnomedicinal use of wormwood in Serbia, collected data in the field.

Gender		Age group		Education	
Male	38 (32.48%)	20–40	15 (12.82%)	Primary	14 (11.96%)
Female	79 (67.52%)	40–60	67 (57.26%)	Secondary	56 (47.86%)
		60–80	35 (29.91%)	High	47 (40.17%)
Total informants	117

Ethnomedicinal use	Citation for particular use	Plant part used (number of reports)	Preparation methods	Fidelity level (%)	
No medicinal use	29	—	—	—	
Aperitif, appetizer	46	24–leaves	Alcoholic solution	52.27	
		22–herb			
Gastrointestinal disorders	35	35–herb	Alcoholic solution; boiled as tea	39.77	
Wound healing	24	11–leaves	Direct application of plant parts on wounded skin; lard coating with dry flowers	27.27	
		10–herb			
		3–flowers			
Coughs	21	21–herb	Tea; alcoholic solution	23.86	
Improvement of memory	7	6–roots	Alcoholic solution; chewing	7.95	
		1–herb			
Preventive	5	5–herb	Tea	5.68	

**Table 2:** Anticandidal activity of *A. absinthium* L. ethanolic extract.

Yeasts	*A. absinthium*		Ketoconazole	
	MIC (mg/mL)	MFC (mg/mL)	MIC (*µ*g/mL)	MFC (*µ*g/mL)
*C. albicans* 475/15	0.5	1.0	3.2	6.4
*C. albicans* 13/15	0.5	1.0	1.6	51.2
*C. albicans* 17/15	0.5	1.0	1.6	51.2
*C. krusei* H1/16	1.0	2.0	1.6	3.2
*C. glabrata* 4/6/15	1.0	2.0	1.6	6.4
*C. albicans* ATCC 10231	1.0	2.0	1.6	6.4
*C. tropicalis* ATCC 750	0.5	1.0	1.6	6.4
*C. parapsilosis* ATCC 22019	0.5	1.0	3.2	6.4

MIC: minimal inhibitory concentration and MFC: minimal fungicidal concentration.

**Table 3:** Compounds identified in *Artemisia absinthium*.

No.	*t*_R_, min	Compound name	Molecular formula, [M–H]^–^	Calculated mass, [M–H]^–^	Exact mass, [M–H]^–^	Δ ppm	MS^2^ fragments, (% Base peak)	MS^3^ fragments, (% base peak)	MS^4^ fragments, (% base peak)	References
1	3.75	Dihydroxybenzoic acid hexoside	C_13_H_15_O_9_^–^	315.07216	315.07153	2.00	**153** (100), 152 (50), 109 (15), 108 (10)	**109** (100)	—	Melguizo-melguizo et al., [32]

2	4.38	Syringic acid hexoside	C_15_H_19_O_10_^–^	359.09837	359.09778	1.64	**197** (100), 182 (10)	**182** (100), 153 (20)	—	Klick and Herrmann, [28]

3	5.23	5-O-Caffeoylquinic acid (chlorogenic acid)^a^	C_16_H_17_O_9_^–^	353.08781	353.08752	0.82	**191** (100), 179 (5)	173 (75), **127** (100), 111 (40), 93 (60), 85 (90)	109 (40), 99 (50), 85 (100)	Han et al., [33]

4	5.77	Esculin^a^	C_15_H_15_O_9_^–^	339.07216	339.07156	1.77	**179** (100), 161 (30), 135 (20)	**135** (100)	—	Han et al., [33]

5	6.17	Feruloylquinic acid	C_17_H_19_O_9_^–^	367.10346	367.10281	1.77	203 (15), 193 (10), **191** (100), 173 (5)	173 (25), **127** (100), 111 (40), 93 (40), 85 (80)	—	Han et al., [33]

6	6.44	Quercetin 3-O-(6″-rhamnosyl)glucoside (Rutin)^a^	C_27_H_29_O_16_^–^	609.14611	609.14545	1.08	343 (5), **301** (100), 300 (30), 271 (10), 255 (5)	273 (25), 257 (20), **179** (100), 151 (75)	151 (100)	Han et al., [33]

7	6.83	Kaempferol 7-O-(6″-rhamnosyl)hexoside	C_27_H_29_O_15_^–^	593.15119	593.15015	1.75	327 (10), 286 (20), **285** (100), 257 (5)	267 (40), **257** (100), 241 (30), 229 (40), 213 (30)	255 (10), 239 (30), 229 (100), 163 (40)	Hoffmann and Harrmann, [27]

8	6.92	Isorhamnetin 3-O-(6″-rhamnosyl)glucoside (Narcissin)^a^	C_28_H_31_O_16_^–^	623.16176	623.16077	1.59	316 (15), 315 (100), 300 (30), 271 (20), 255 (10)	301 (5), **300** (100), 287(5), 272(5), 255(5)	272 (100), 271(90), 255 (50), 243 (10), 166 (5)	Hoffmann and Harrmann, [27]

9	7.00	Apigenin 8-*C*-[6“-(3-hydroxy-3-methylglutaryl)]hexoside	C_27_H_27_O_14_^–^	575.14063	575.14032	0.54	557 (10), 513 (15), **431** (100), 341 (80), 311 (70)	341 (10), **311** (100)	283 (100)	—

10	7.00	Spinacetin 3-O-(6″-rhamnosyl)hexoside	C_29_H_33_O_17_^–^	653.17232	653.17206	0.40	346 (20), **345** (100), 330 (40), 302 (15), 287 (20)	**330** (100), 305 (5)	315 (10), 302 (100), 287 (30), 273 (5), 166 (5)	Hoffmann and Harrmann, [27]

11	7.11	Dicaffeoylquinic acid isomer 1	C_25_H_23_O_12_^–^	515.11950	515.11975	-0.49	**353** (100)	**191** (100), 179 (40), 173 (5), 135 (10)	173 (95), 171 (50), 127 (90), 111 (30), 85 (100)	Han et al., [33]

12	7.20	Isorhamnetin 3-O-glucoside^a^	C_22_H_21_O_12_^–^	477.10385	477.10339	0.96	357 (15), **315** (100), 314 (90), 299 (20), 285 (15)	**300**(100), 286(5), 272(10)	283 (5), 272 (100), 271 (60), 255 (40), 243 (10)	Hoffmann and Harrmann, [27]

13	7.25	Dicaffeoylquinic acid isomer 2	C_25_H_23_O_12_^–^	515.11950	515.11932	0.35	**353** (100), 335 (5), 299 (10), 255 (5), 203 (10)	**191** (100), 179 (65), 173 (60), 135 (20)	173 (80), 171 (20), 127 (80), 111 (20), 85 (100)	Han et al., [33]

14	7.28	Spinacetin 3-O-hexoside	C_23_H_23_O_13_^–^	507.11441	507.11395	0.91	492 (60), **345** (100), 344 (80), 330 (20), 329 (60)	**330** (100), 302 (5), 286 (10)	—	Hoffmann and Harrmann, [27]

15	7.43	Chrysoeriol 7-O-hexoside	C_22_H_21_O_11_^−^	461.10894	461.10904	−0.22	446 (10), 300 (10), **299** (100), 284 (10)	**284** (100)	256 (100)	Benyahia et al., [31]

16	7.50	Rosmarinic acid^a^	C_18_H_15_O_8_^–^	359.07724	359.07672	1.45	223 (10), 197 (30), 179 (40), **161** (100), 133 (10)	**133** (100)	105 (100)	Sahin et al., [30]

17	9.52	Apigenin^a^	C_15_H_9_O_5_^−^	269.04554	269.04529	0.93	269 (60), **225** (100), 201 (30), 151 (70), 149 (50)	210 (10), 197 (50), 196 (20), 183 (40), **181** (100)	—	Olennikov et al., [34]

18	9.73	Chrysoeriol^a^	C_16_H_11_O_6_^–^	299.05611	299.05576	1.17	285 (10), **284** (100)	284 (20), **256** (100)	256 (40), 228 (100), 160 (40)	Olennikov et al., [34]

19	11.43	Eupatorin	C_18_H_15_O_7_^–^	343.08233	343.08160	2.13	329 (10), **328** (100)	314 (10), **313** (100), 285 (5)	298 (100), 285 (10), 270 (15)	Rashid et al., [61]

20	11.67	Casticin	C_19_H_17_O_8_^–^	373.09289	373.09250	1.05	359 (10), **358** (100)	**343** (100)	328 (100), 315 (15), 300 (30), 299 (20), 284 (10)	Han et al., [33]

21	11.75	Kaempferide^a^	C_16_H_11_O_6_^–^	299.05611	299.05573	1.27	285 (10), **284** (100)	255 (20), 240 (15), 228 (20), 164 (25), **151** (100)	106 (100), 83 (10), 65 (5)	Lai et al., [29]

^a^Confirmed using available standards, all the other compounds were identified based on HRMS data. Bold numbers are peaks which were further fragmented in MS^3^ and MS^4^ experiment.

**Table 4:** Antibacterial activity of *A. absinthium* L. ethanolic extract.

Bacteria	*A. absinthium* (mg/mL)		Streptomycin (*µ*g/mL)	
	MIC	MBC	MIC	MBC
*Micrococcus luteus* (dT_9/2)	0.5	1.0	6.2	12.5
*Rothia mucilaginosa* (oT_22/2)	0.5	1.0	12.5	25
*Streptococcus agalactiae* (oT_20/1)	1.0	2.0	3.1	6.2
*Streptococcus anginosus* (oT_26)	2.0	4.0	3.1	6.2
*Streptococcus dysgalactiae* (oT_21/2)	0.5	1.0	6.2	12.5
*Streptococcus oralis* (oT_5)	1.0	2.0	12.5	25
*Streptococcus parasanguinis* (oT_3)	0.5	1.0	3.1	6.2
*Streptococcus pyogenes* (dT_14)	2.0	4.0	3.1	6.2
*Streptococcus pyogenes* (IBR S004)	1.0	2.0	14.0	28.0
*Streptococcus salivarius* (dT_12)	0.2	0.5	6.2	12.5
*Streptococcus salivarius* (IBR S006)	1.0	2.0	3.8	7.6
*Enterococcus faecalis* (IBR E001)	0.5	1.0	50.0	100.0
*Staphylococcus hominis* (oT_14/2)	2.0	4.0	37.5	75.0
MRSA (IBRS MRSA 011)	4.0	8.0	100	>100
*Listeria monocytogenes* (NCTC 7973)	4.0	8.0	50.0	100
*Enterobacter cloacae* (oT_18)	0.5	1.0	37.5	75
*Enterobacter cloacae* (ATCC 35030)	0.5	1.0	25.0	50.0
*Stenotrophomonas maltophilia* (A_12)	2.0	4.0	37.5	75.0
*Pseudomonas aeruginosa* (IBRS P001)	1.0	2.0	50.0	100.0
*Yersinia enterocolitica* (ATCC 23715)	1.0	2.0	10.0	20.0
*Klebsiella pneumoniae* (ATCC 13883)	1.0	2.0	5.0	10.0
*Salmonella enterica* (ATCC 13311)	1.0	2.0	50.0	100.0
*Escherichia coli* (IBRS E003)	4.0	8.0	100	>100
*Escherichia coli* (ATCC 35210)	1.0	2.0	50.0	100

MIC: minimal inhibitory concentration and MBC: minimal bactericidal concentration. Results are in mg/mL.

## Data Availability

The datasets used and analyzed in the current study are included within the article.
